# Alcohol Metabolizing Enzymes, Microsomal Ethanol Oxidizing System, Cytochrome P450 2E1, Catalase, and Aldehyde Dehydrogenase in Alcohol-Associated Liver Disease

**DOI:** 10.3390/biomedicines8030050

**Published:** 2020-03-04

**Authors:** Yanchao Jiang, Ting Zhang, Praveen Kusumanchi, Sen Han, Zhihong Yang, Suthat Liangpunsakul

**Affiliations:** 1 Division of Gastroenterology and Hepatology, Department of Medicine, Indiana University School of Medicine, Indianapolis, IN 46202, USA; yancjian@iu.edu (Y.J.); tz12@iu.edu (T.Z.); pkusuman@iu.edu (P.K.); senhan@iu.edu (S.H.); 2 Roudebush Veterans Administration Medical Center, Indianapolis, IN 46202, USA

**Keywords:** alcohol metabolizing enzyme, cytochrome P450 2E1, and alcohol-associated liver disease

## Abstract

Once ingested, most of the alcohol is metabolized in the liver by alcohol dehydrogenase to acetaldehyde. Two additional pathways of acetaldehyde generation are by microsomal ethanol oxidizing system (cytochrome P450 2E1) and catalase. Acetaldehyde can form adducts which can interfere with cellular function, leading to alcohol-induced liver injury. The variants of alcohol metabolizing genes encode enzymes with varied kinetic properties and result in the different rate of alcohol elimination and acetaldehyde generation. Allelic variants of these genes with higher enzymatic activity are believed to be able to modify susceptibility to alcohol-induced liver injury; however, the human studies on the association of these variants and alcohol-associated liver disease are inconclusive. In addition to acetaldehyde, the shift in the redox state during alcohol elimination may also link to other pathways resulting in activation of downstream signaling leading to liver injury.

## 1. Introduction

Alcohol associated liver disease (ALD) is one of the major causes of chronic liver disease worldwide [1,2]. The risk of ALD development is known to be associated with the quantity of alcohol (beverages containing ethanol) consumed [3,4]. Early studies suggested that a long-term consumption of more than 80 g per day was correlated with increased risk of cirrhosis, but subsequent estimates of the harmful drinking threshold have been below this level, especially for women [5,6]. In another study, 13,285 subjects were prospectively followed for 12 years to determine the association between the risk for development of future liver disease and self-reported alcohol consumption [7]. The patients were asked in multiple-choice form on the type of alcoholic beverages they consumed, wine (glasses), beer (bottles), or spirits (units) and frequency (i.e., daily, weekly, monthly, and hardly/never). If daily alcohol intake was reported, the average number of beverages of each type per week was recorded. In this study, one beverage equals to 12 g of alcohol [7]. There was no apparent risk of alcohol-induced liver injury if the range of alcohol consumption was between 1 to 6 beverages per week (12–72 g per week); however, drinking beyond this level (7 to 13 beverages (84–156 g) per week for women and 14 to 27 beverages (168–324 g) per week for men) resulted in a significant rise in the relative risk of alcohol associated liver disease [7]. In addition to the quantity of alcohol consumption, studies also suggested that the risk of ALD may also depend on the patterns and types of alcohol intake independently of the level of alcohol consumption [8]. Red wine drinkers may have a lower risk of ALD compared to other beverage drinkers [9]. It is currently unknown whether this is due to an effect of the red wine per se or to confounding protective lifestyle factors. Of importance, it is difficult to systematically study the relationship between the quantity of alcohol consumption and the development of ALD because data collection always involves rough estimate, differences in drinking patterns among studies, and inconsistencies in data collection tools. ALD represents a range of pathological changes ranging from steatosis, alcoholic steatohepatitis, advanced fibrosis, and cirrhosis [1,10]. Alcoholic steatosis can occur in patients who consumed 120–150 g of alcohol per day for 2–3 weeks [11]. Most cases develop macrovesicular steatosis primarily in zone 3 or throughout the liver in severe cases [12]. This condition is reversible upon abstinence [13]. Hepatic steatosis was thought to be a benign condition; however, a subset of patients can progress to fibrosis or cirrhosis in 10 years, especially if the drinking continues [14].

Only a group of patients with excessive alcohol use develop alcoholic hepatitis, a severe manifestation of ALD with high morbidity and mortality [15,16,17]. Approximately, 10% to 20% of patients with alcoholic hepatitis are likely to progress to cirrhosis annually, and up to 70% of patients with alcoholic hepatitis ultimately will become cirrhotic [12]. Abstinence is essential and has the impact on the survival [15,18]. Overall, 15% to 20% of excessive drinkers will progress to advanced liver disease and cirrhosis [19]. Once developed, patients with alcoholic cirrhosis have clinical presentations and complications similar to those with other types of cirrhosis. Of importance, mortality due to alcoholic cirrhosis has been shifted towards a younger population between 25 and 34 years old [20]. There are also important gender differences in ALD pathogenesis. At any given level of alcohol consumption, women have a higher likelihood of developing ALD than men [5]. The mechanism is likely due to lower volume of distribution in women leading to higher blood alcohol concentrations per unit of alcohol consumed [21]. Additionally, recent studies also showed the role of hormone like estrogen which can increase gut permeability and endotoxemia leading to the increase in the production of inflammatory cytokines by Kupffer cells and risk of ALD [22]. Some of the enzymes involved in alcohol metabolism are genetically polymorphic leading to the alteration in enzymatic properties and varying amounts of acetaldehyde generation after alcohol consumption [23]. The roles of these enzymes in association with ALD are the subject of this overview.

## 2. Alcohol Metabolism

When alcohol is ingested, a small amount is immediately metabolized in the stomach. Most of the remaining alcohol is subsequently absorbed from the gastrointestinal tract, primarily the stomach and upper small intestine [24]. After the absorption, alcohol is transported to the liver through the portal vein, where the alcohol concentration is significantly higher than that measured from the peripheral blood [25]. Absorption rate of ethanol is a first-order kinetic process in proportion to the concentration of alcohol in the stomach. Once the gastric alcohol concentration decreases, the rate of absorption slows down [26]. After the initial passage through the liver; the remainder of the ingested alcohol enters systemic circulation and is distributed throughout the body’s tissues. Once the rate of absorption is equal to the rate of alcohol metabolism (approximately 10–30 mg% per hour), the peripheral blood alcohol concentration reaches a maximum point and starts to decline as alcohol is eliminated from the body [27].

**Figure 1:**
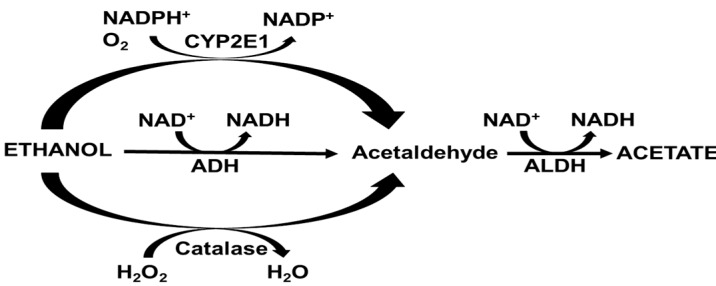
The major pathways of acetaldehyde formation after alcohol ingestion, alcohol dehydrogenase (ADH), cytochrome P450 2E1 (CYP2E1), catalase, and aldehyde dehydrogenase (ALDH) are demonstrated with their cofactors, and substrates.

Liver is the major organ which metabolizes more than 90% of ingested alcohol into acetaldehyde through several enzymatic and non-enzymatic mechanisms [28,29]. Many of the effects of ethanol are mediated by its byproduct acetaldehyde, which is mainly generated by alcohol dehydrogenase (ADH), cytochrome P450 2E1 (CYP2E1), and catalase (Figure 1). Acetaldehyde is subsequently converted to acetate by aldehyde dehydrogenases (ALDH), which is released from the liver and metabolized by muscle and heart [29]. The rate of ethanol metabolism by alcohol metabolizing enzymes may be critical in determining its toxicity because of the toxic intermediates.

## 3. Alcohol Metabolizing Enzymes

### 3.1. Alcohol Dehydrogenase (ADH)

ADH is a zinc-dependent enzyme located in the cytosol. It uses NAD^+^ as a cofactor and is responsible for the majority of alcohol oxidation in the liver [30,31]. Human ADHs are categorized into five classes based on their structural and kinetic characteristics [23,29]. These enzymes are dimeric and identified by Greek letters with a molecular weight of 40kDa per subunit [23,29]. Under physiological conditions, the main isoenzymes involved in human ethanol metabolism are ADHs from classes I, II, and IV [23,29]. 

Class I ADH contains α, β, and γ isoenzymes. These enzymes have a low Michaelis constant (Km) < 5 mM for ethanol and therefore play a significant role in ethanol metabolism [23]. Class I ADH is highly abundant in the liver and is sensitive to inhibition by pyrazole derivatives [32]. Class I ADH consists of three genes, *ADH1A, ADH1B*, and *ADH1C*, making up gene clusters located on chromosome 4q21-23, spanning about 80 kb [33]. Among these genes, polymorphisms with physiological significance exist in the *ADH1B* and *ADH1C* loci [23]. These variants result in the difference in their efficiency for ethanol oxidation. People of different racial backgrounds inherit different sets of ADH isoenzymes [29,34,35]. African Americans who carry a copy of the ADH1B*3 gene, encoding an enzyme with high maximum velocity, have a somewhat faster alcohol metabolism rate [36,37]. Class II ADH (π ADH) has a higher Km for ethanol and is less sensitive to pyrazole inhibition than class I enzymes [38]. Because of its Km, its role in ethanol metabolism is less than that of Class I ADH [39]. Class III ADH is ubiquitous and it can metabolize longer chain alcohols as well as ω-oxidation of fatty acid [40]. The class IV ADH (µ-ADH, for the mucosal isozyme or σ-ADH, for the stomach isoenzyme) has been found in the esophagus and stomach and is responsible for alcohol first pass metabolism [41]. Among ADHs, σ-ADH has the highest *V*max and is very active towards retinol. This may be pertinent to its expression at the epithelia, which are dependent on retinol for their integrity [42]. The kinetic properties of each ADH class, population and tissue distribution are shown in Table 1. 

Tissue distribution of ADH: *ADH* mRNAs are expressed in a variety of tissues with high levels of class I ADH in the liver, stomach, kidney, and colon, and with lower levels in lung, small intestine, brain, and muscle [43]. Of importance, class I ADH is also found in blood vessels, which may be linked to alcohol-induced flushing. Class II ADH was found in the liver and duodenum [43]. σ-ADH is primarily found in the gastric mucosa, though its expression is absent in approximately one-third of the Asians population [44]. Microorganisms can produce alcohol [45,46] and express numerous forms of alcohol dehydrogenase, which can contribute to the formation of acetaldehyde in the gastrointestinal tract, or wherever microbial overgrowth occurs [47,48].

Regulation of ADH expression: The promotors of hepatic ADH1 can interact with ubiquitous transcription factors (i.e., upstream stimulatory factor [USF], TATAA binding factors and CAAT box-binding transcription factor/nuclear factor-1 [CTF/NF-I]), and tissue-specific factors (i.e., CCAAT-enhancer binding proteins [C/EBPα and β] hepatocyte nuclear factor 1 [HNF-1], and D-box binding protein [DBP]) [49,50,51,52]. An HNF-1 site was found to serve as a key regulator for all three class I genes [53]. Upstream regions of *ADH1* genes are also found to be the binding sites for retinoic acid, glucocorticoid and thyroid hormones [23,54,55,56]. Hepatic ADH activity also depends on the route of alcohol administration. While the quantity of ethanol consumed from liquid diets did not modify hepatic ADH activity, higher doses of ethanol administered by intragastric infusion induced hepatic ADH activity due to the induction of the transcription factor C/EBPβ and suppression of C/EBPγ and a truncated, inhibitory form of C/EBPβ [57]. Additionally, intragastric ethanol infusion can induce hepatic Class I *ADH* mRNA, protein, and activity levels by 3.9-, 3.3-, and 1.7-fold, respectively through transcription factor USF due to the increase in endotoxin in the portal system [57,58].

Level of alcohol concentration and ADH: Because of the low Km of Class I ADH, this enzyme is saturated with any types of drinking patterns once blood alcohol concentration >15–20 mg% [27]. While class I ADH with low Km for ethanol is active even at a low alcohol concentration, those with high Km (i.e., σ-ADH) are more active when blood ethanol concentrations are high or in tissues of the upper gastrointestinal tract that are directly exposed to beverage ethanol. Modelling of ethanol oxidation indicated that ethanol metabolism by ADH is regulated by the quantity of ADH enzymes, the byproduct acetaldehyde, and the NADH to NAD^+^ ratio in the cytosol [59,60].

### 3.2. Microsomal Ethanol Oxidizing System

In addition to ADH, ethanol can be oxidized by a microsomal ethanol oxidizing system (MEOS) involving primarily the cytochrome P450 2E1 (CYP2E1) [61]. Two other cytochromes contribute to the MEOS, albeit to a lesser extent, CYP1A2 and CYP3A4 [62,63,64]. CYP2E1 is associated with NADPH-cytochrome P450 reductase in the endoplasmic reticulum and reduces oxygen molecule to water when ethanol is oxidized to acetaldehyde [61]. It has a low ethanol catalytic efficiency with the Km for ethanol ~10 mM when compared to ADH and therefore is responsible for a minor role and accounting for approximately 10% of ethanol metabolism [65]. However, CYP2E1 may assume a greater role at high blood alcohol concentrations. The levels of CYP2E1 are induced by chronic alcohol consumption (more detail in section below) notably in the perivenular zone leading to the increase in alcohol elimination rate among excessive drinkers [23]. In addition, it is induced in diabetes, high fat diet, and fasting, which may relate to its ability to oxidize the ketone body acetone [66,67,68,69]. CYP2E1 is a major contributor of oxidative stress in the hepatocytes by generating several reactive oxygen species (ROS) such as hydrogen peroxide (H_2_O_2_), hydroxyethyl radical (HER·), hydroxyl radical (OH^−^) and superoxide (O_2_^−^) [64,67].

Regulation of CYP2E1 expression: The human *CYP2E1* gene spans 11 kb, contains 9 exons, and contains a typical TATAA box [70]. Its expression is regulated at the transcriptional (mRNA) level by ethanol concentration [71] and at the post translational level by protein stabilization and reducing the proteasomal degradation [64]. The expression of *CYP2E1* can also be induced by cytokines such as interleukin-4 [72]. *CYP2E1* can also be regulated by hsa-miR-214–3p [73]. In a HepG2-derived *CYP2E1* over-expression cell model, hsa-miR-214-3p showed strong suppression of *CYP2E1* expression by targeting the coding region of its mRNA transcript [73]. Treatment with hsa-miR-214-3p mimics partially blocked ethanol-dependent increases in *CYP2E1* mRNA and protein levels in HepG2 cells [73].

Level of alcohol concentration and CYP2E1: Since CYP2E1 has a high Km for ethanol, it will generate acetaldehyde at a high level of ethanol concentrations [23]. CYP2E1 plays a more important role in alcohol metabolism when high blood concentration is reached. Another important aspect of CYP2E1 is that binge drinking can induce its activity, making it more effective in alcohol metabolism at its higher blood concentration [74]. Additionally, the increase in enzyme activity is the underlying mechanism to explain faster alcohol clearance among excessive drinkers [63]. Unlike ADH, it does not appear that CYP2E1 is inhibited by acetaldehyde; in fact, CYP2E1 can oxidize acetaldehyde to acetate, but not in the presence of ethanol [23]. Because CYP2E1 also metabolizes several medications, excessive drinkers with the induction in CYP2E1 activity will demonstrate increased metabolic rates for those medications which may lead to adverse consequences [75].

### 3.3. Catalase

The peroxisomal catalase, a tetrameric, heme-containing enzyme, converts hydrogen peroxide (H_2_O_2_) to oxygen and water [76]. Catalase can also oxidize ethanol to acetaldehyde as the end product in an H_2_O_2_-dependent fashion, though this is not a key pathway for ethanol elimination [23,77].

Tissue distribution of catalase: Catalase is ubiquitously expressed in almost all tissues. Catalase is also expressed by colonic floras which may lead to acetaldehyde production in the lower gastrointestinal tract [78,79].

Regulation of catalase expression: In rodents, catalase gene is a single-copy gene with 33 kb. The promoter region of this gene lacks a TATAA box and an initiator consensus sequence [23]. It contains multiple transcription initiation sites as well as multiple CCAAT boxes and GC boxes [23]. The increase in catalase activity was reported after chronic ethanol feeding [80]. The rat catalase promoter contains a peroxisome proliferator responsive element (PPRE), therefore it can be induced by peroxisome proliferators [81].

Level of alcohol concentration and catalase: Catalase activity relies on the cellular level of H_2_O_2_. The ability of catalase to metabolize ethanol may be increased under oxidative stress with the increase in cellular H_2_O_2_ production [82]. This was observed with perfused rat liver, when fatty acids were added and the process of peroxisomal β oxidation lead to the generation of H_2_O_2_ and oxidation of ethanol. This raises the possibility that under conditions of oxidant stress (and H_2_O_2_ production) catalase-mediated ethanol oxidation may be increased [23].

### 3.4. Aldehyde Dehydrogenase (ALDH)

The second step of ethanol metabolism is the process to convert acetaldehyde to acetate by alcohol dehydrogenase (ALDH). ALDH enzymes use NAD^+^ as a cofactor during the oxidization of acetaldehyde but are expressed in a wider range of tissues than the ADH isoenzymes [83]. In addition to its role in elimination of acetaldehyde, ALDH also plays a critical role in the detoxification of lipid peroxidation byproduct such as 4-hydroxynonenal (4-HNE) which is generated during oxidative stress [84]. There are two major ALDH isoforms, cytosolic and mitochondrial, encoded by the aldehyde *ALDH1* and *ALDH2* genes, respectively. Of the two isoforms, mitochondrial ALDH2 plays the central role in human acetaldehyde metabolism because of its submicromolar Km for acetaldehyde [29]. Tissue distribution and Km for acetaldehyde of human ALDHs are shown in Table 2.

Regulation of ALDH2 expression: ALDH2 expression can be regulated at the transcriptional level [85]. *ALDH2* gene promoter contains a nuclear receptor response element that is bound by retinoic acid receptors, peroxisome proliferator activated receptors (PPARs), hepatocyte nuclear factor 4 (HNF4), and members of the chicken ovalbumin upstream promoter transcription factor (COUP-TF) family [85,86,87]. ALDH2 activity can also be modified through post-translational regulation by serine/threonine phosphorylation through epsilon protein kinase C (εPKC) [84]. Additionally, the activation of ALDH2 by ethanol has been shown to be mediated by epigenetic regulation through hyperacetylation of *ALDH2* gene by sirtuin 3 (SIRT3) inactivation [88].

### 3.5. Other Minor Pathways for the Generation of Acetaldehyde

Cytosolic fractions of xanthine oxidoreductase such as hypoxanthine, xanthine, and caffeine can oxidize ethanol to acetaldehyde [89]. This process can be inhibited by allopurinol and not by pyrazole [89]. Ethanol can also be metabolized to acetaldehyde and 1-hydroxyethyl radical by cytochrome P450 reductase [90]. There are other reports that other oxidant species (hydroxyl radical) formed non-enzymatically might be able to oxidize ethanol to acetaldehyde. In addition, acetaldehyde can be formed during the degradation of threonine, putatively by threonine aldolase [23,29].

## 4. Genetic Polymorphisms of Alcohol-Metabolizing Enzymes and ALD

ADH: Because of the differences in the enzymatic activity to metabolize alcohol to acetaldehyde, it has been postulated that those with alleles encoding for higher enzymatic activity such as ADH1B*2 and ADH1C*1 alleles, are at increased risk of developing ALD and modifying the risk for alcohol dependence due to higher acetaldehyde exposure at any given alcohol concentration [91,92]. ADH1B*2 allele has been shown to reduce the occurrence of alcohol abuse especially in population with high prevalence such as Jewish and Asian populations [29]. Several other studies also found similar results on the higher frequency of ADH1B*2 among moderate or non-drinkers compared to heavy drinkers [93,94,95]. The reason behind these findings is likely due to the development of unpleasant side effects associated with the faster accumulation of acetaldehyde after alcohol consumption [96], though the levels of venous acetaldehyde concentration in those with and without this allele have not previously shown to be different [97,98]. A recent study from Japan found that those with slow metabolizing ADH1B enzyme increase susceptibility to hepatic steatosis in men with alcohol dependence [99]. However, to date, the results on the association between genetic variants of ADH and ALD have been inconsistent, owing to the difference in the ethnicities of the study cohorts and limitations in sample size, as well as the low allele frequency of ADH1B*2 among Caucasians [92].

CYP2E1: The *CYP2E1 c1* allele is located in the 5’-flanking region of the *CYP2E1* gene while in vitro transcriptional assays showed the *c2* allele to be more active enzyme [100,101]. The allele frequency of *CYP2E1* variants is varied depending on ethnicity, the *c2* variant is found at a higher frequency in East Asians (~25–35%) than that in Caucasians (~2–8%) [23,102]. In contrast to ADH, CYP2E1 is inducible and its activity can increase significantly after alcohol consumption [23], causing a higher level of reactive oxygen species and acetaldehyde [103]. The allele frequency of *c2* variant is higher than that of *c1* in patients with ALD, compared with random population controls [92]. However, the association between *CYP2E1* variants and ALD is inconclusive [92].

Catalase: Transcription of the catalase gene is influenced by the -262 C/T and -844 A/G polymorphisms. A common polymorphism -262 C/T in the promoter region has been found to be associated with altered catalase activities and is associated with the susceptibility to alcohol dependence [104,105]. To date, there is no convincing evidence on the role of catalase polymorphism and ALD.

ALDH: Similar to ADH and CYP2E1 variants, polymorphisms in the *ALDH2* demonstrate a vital role in regulating ALDH2 activity and are hypothesized to alter genetic susceptibility to alcohol dependence and alcohol-induced liver diseases. A variant at exon 12 predicts lysine at residue 504 instead of glutamic acid [106]. The common form of the SNP (rs671) (504glu) encodes the glu (G) allele (previously referred to as the ALDH2 *1 allele); the 504lys (A, formerly ALDH2 *2 and 487lys) allele produces an inactive isozyme and thus limits its activity to convert acetaldehyde into acetate [106,107]. Individuals with the lys allele tend to have the accumulation of acetaldehyde after alcohol ingestion with the peak acetaldehyde concentration approximately 6- to 19-fold higher in those with heterozygotes or homozygotes for 504lys allele than those with common allele [97]. These patients commonly experience unpleasant side effects such as nausea, vomiting, or flushing after alcohol ingestion [108,109]. ALDH2 is inhibited by disulfiram (Antabuse) and consumption of alcohol while taking disulfiram or a number of other drugs such as metronidazole results in the accumulation of acetaldehyde, producing vasodilation, facial flushing, tachycardia, nausea, and vomiting [37]. Variants in genes encoding *ALDH2* are important determinants of risk for alcohol abuse or dependence and to a lesser extent for cirrhosis, especially in Asian populations [92]. Previous studies have shown that the inactive *ALDH2 504lys* allele occurred mainly in Asians [29,37]. Most patients with alcohol abuse or dependence with inactive *ALDH2* alleles were heterozygous while homozygous lys/lys was rarely found, although it was often observed in controls [106]. These findings are consistent with what we observed in our study [110]. *ALDH2 504lys* allele is reported to be less prevalent in patients with alcoholic cirrhosis than that in controls [110]. Depending on the geographic region, the allele frequency for 504lys has been reported to be up to 40% among East Asian population [111], however, its frequency among patients with ALD is around 1.0%–11.6%, depending on the population being studied [106]. Taken together, there are several evidence linking the genetic variants of alcohol metabolizing enzyme genes with the risk of alcoholism/alcohol dependence; however, the association of these variants and ALD is still inconclusive.

## 5. Ethanol Metabolism and Its Association in ALD Pathogenesis

### 5.1. Redox State Alterations and ALD

Ethanol metabolism by alcohol metabolizing enzymes leads to the changes in intracellular redox state which may underlie the pathogenesis of ALD. One of the earliest responses to excessive alcohol use is the development of hepatic steatosis secondary to the accumulation of triglyceride droplets in the hepatocytes [10]. One of the pathophysiological mechanisms leading to hepatic steatosis is the imbalance between triglyceride synthesis and the ability to oxidize or export it in the form of very low-density lipoprotein (VLDL) particles [10]. Increasing intracellular NADH levels secondary to ethanol metabolism from ADH and ALDH inhibit fatty acid oxidation [112]. Additionally, the induction of a few enzymes involved in fatty acid synthesis as well as increase in the intracellular level of fatty acids and L-glycerol-3-phosphate result in the increase in the synthesis of triglycerides [10,113]. The change in the intracellular redox state leading to triglyceride accumulation has been confirmed in cell lines with stably expressed ADH. These cell lines can oxidize ethanol resulting in the alterations in intracellular redox potential as indicated by an increase in the lactate/pyruvate ratio. More importantly, they can accumulate large amounts of triglyceride upon alcohol treatment [114].

The alterations in the redox state, among several mechanisms, may interfere with sirtuin-1 (SIRT-1), a nicotinamide adenine dinucleotide (NAD+, NADH)-dependent class III histone deacetylase [115,116]. Ethanol-mediated SIRT1 inhibition may lead to hyper-acetylation of a set of molecules including histone H3, transcription factors such as sterol regulatory element-binding protein (SREBP) and peroxisome proliferator-activated receptor alpha (PPARα) [116]. This subsequently leads to the alterations in several downstream pathways which are involved in inflammatory responses and lipid metabolism leading to the development of ALD (see more detail in [116]).

### 5.2. Role of Oxidative Stress

As previously mentioned, CYP2E1 is induced by chronic alcohol use and is “leaky,” in that electrons transferred to it from CYP450 reductase can eventually be transferred to oxygen molecule forming superoxide [67,117]. The consequence of intracellular oxidative stress is lipid peroxidation [118], a reaction of lipid radical formation that follows the removal of an electron from polyunsaturated fatty acid (PUFA) [118,119]. The lipid free radical then form a lipid hydroperoxide with the addition of oxygen molecule, and ultimately decomposition to form reactive aldehydes such as 4-hydroxynonenal (4-HNE) and malondialdehyde (MDA) [120,121,122,123]. ALD has been shown to be associated with increase in lipid peroxidation, formation of 1-hydroxyethyl radical and lipid radicals, and decrease in hepatic antioxidants, especially glutathione [67,82,124]. In addition to the role of CYP2E1-induced oxidative stress, alcohol can also acetylate mitochondrial superoxide dismutase and impact its antioxidant ability [125]. Changes in diets that either promote or reduce oxidative stress have an impact on the degree of liver injury. When PUFA were substituted with either medium-chain triglycerides or saturated fats, the extent of alcohol-induced liver injury was ameliorated because of the reduction in lipid peroxidation [126,127]. CYP2E1 may be an important regulator of liver injury in obese patients with excessive alcohol use. Obesity is an independent risk factor for hepatic steatosis, alcoholic hepatitis, and cirrhosis among excessive drinkers [8,128]. Obesity and alcohol synergistically enhance the degree of liver injury [129,130]. Several studies indicate combined pathological effects of alcohol and obesity or fatty acid levels, respectively, on hepatocellular lipid accumulation and injury as well as hepatic inflammation, fibrosis, and cancerogenesis [130]. The induction of CYP2E1 is commonly observed in patients with excessive alcohol use and interestingly it can also be activated in the presence of obesity and metabolic syndrome by increasing levels of fatty acids [63,131,132]. The co-activation of CYP2E1 by both ethanol and endogenous substrates such as fatty acids, can augment the severity and accelerate the progression of liver injury [132,133]. In fact, a recent study in a cohort of 233 patients with alcoholic hepatitis from the United Kingdom and United States demonstrated that obesity is common in AH and associated with a greater than two-fold increase in short-term mortality [134].

### 5.3. Role of Protein Adduct Formation

Protein adducts formation during alcohol-induced liver injury are covalent linkages developed between amino acid side chains of liver proteins and compounds such as acetaldehyde, and byproducts of lipid peroxidation 4-HNE and MDA [135]. Once generated, acetaldehyde can form the covalent linkages with amino acid side chains of hepatic proteins to form acetaldehyde adducts. These adducts are located primarily in the perivenous region and develop early in the process of alcohol-induced liver injury [136]. Once the disease progresses, they are prevalent in areas of inflammation and fibrosis [137,138]. It has been well documented that these adducts elicit an immune response and can interfere with the functions of proteins especially at the lysine residues [139], such as lysine-dependent enzymes as well as cytoskeletal proteins [140]. There are reports that show acetaldehyde adducts can impair microtubule function leading to defects in protein transport which in turn attributes to endoplasmic reticulum stress in alcohol-induced liver injury [140].

## 6. Conclusions

ADH, CYP2E1, and catalase can generate acetaldehyde after alcohol ingestion. The ability of acetaldehyde to alter cellular function will depend on the enzymatic activity and acetaldehyde formation. Because of the differences in the capacity to metabolize alcohol to acetaldehyde, it has been suggested that individuals with high activity of alcohol metabolizing enzymes are susceptible to alcohol-induced liver injury due to a higher level of acetaldehyde exposure. However, the results on the allelic variants of the genes encoding for ADH, CYP2E1 genes, and ALD are inconclusive. Alcohol metabolizing enzyme, notably CYP2E1, may be an important regulator leading to liver injury especially in obese patients with excessive alcohol use. In addition to acetaldehyde, the shift in the redox state during alcohol elimination may also inter link to other pathways and trigger downstream effects leading to ALD. The knowledge gain from the studies of alcohol metabolism by these enzymes may perhaps open the new avenue for the treatment of ALD [141].

## Figures and Tables

**Table 1:** The kinetic properties of each ADH class, population, and tissue distribution [23,29].

Gene Locus	Subunit	Km for Ethanol (mM)	Vmax (min^−1^)	Major Ethnicity	Tissue Distribution
Class I					
ADH1B*1	β1	0.05	54	Caucasians, African-Americans	Liver, lung
ADH1B*2	β2	0.9	-	Asians	
ADH1B*3	β2	34	-	African-Americans	
ADH1C*1	γ1	1	-	All groups	Liver, stomach
ADH1C*2	γ2	0.63	-	Caucasians	
Class II: *ADH2*	π	34	40		Liver
Class III: *ADH3*	χ	1000	-		Ubiquitous
Class IV: *ADH4*	σ, µ	20	1510		Stomach and esophagus

“-“: no data available.

**Table 2:** Km for acetaldehyde and tissue distribution of human aldehyde dehydrogenase (ALDH) [23,29].

Gene Locus	Km for Acetaldehyde	Tissue Distribution
Class I ALDH1	30 µM	Liver and many other tissues
Class II ALDH2	<1 µM	Low levels in most tissues with the expression highest in liver compared to kidney and muscle
Class III ALDH3	11 mM	Stomach, liver, cornea

## Abbreviations

ALD	Alcohol associated liver disease
ADH	Alcohol dehydrogenase
ALDH	Aldehyde dehydrogenase
C/EBP	CCAAT-enhancer-binding proteins
COUP-TF	Chicken ovalbumin upstream promoter transcription factor
CTF/NF-I	TATAA binding factors and CAAT box-binding transcription factor/nuclear factor-1
CYP	Cytochrome
CYP2E1	Cytochrome P450 2E1
DBP	D-box binding protein
HNE	Hydroxynonenal
HNF	hepatocyte nuclear factor
MDA	Malondialdehyde
MEOS	Microsomal ethanol oxidizing system
PKC	Protein kinase C
NAD	Nicotinamide adenine dinucleotide
NADP	Nicotinamide adenine dinucleotide phosphate
PKC	protein kinase C
PPARs	Peroxisome proliferator activated receptors
PPRE	Peroxisome proliferator responsive element
PUFA	Polyunsaturated fatty acid
ROS	Reactive oxygen species
SIRT	Sirtuin
SREBP	Sterol regulatory element-binding protein
USF	Upstream stimulatory factor
VLDL	Very low density lipoprotein
